# The timing of adrenarche in Maya girls, Merida, Mexico

**DOI:** 10.1002/ajhb.23465

**Published:** 2020-07-09

**Authors:** Sarai M. Keestra, Gillian R. Bentley, Alejandra Núñez‐de la Mora, Lauren C. Houghton, Hannah Wilson, Adriana Vázquez‐Vázquez, Gillian D. Cooper, Federico Dickinson, Paula Griffiths, Barry A. Bogin, Maria Inês Varela‐Silva

**Affiliations:** ^1^ Department of Anthropology Durham University Durham UK; ^2^ Instituto Investigaciones Psicológicas Universidad Veracruzana Mexico; ^3^ Mailman School of Public Health Columbia University USA; ^4^ Telligen Des Moines Iowa USA; ^5^ Childhood Nutrition Research Centre (CNRC) University College London UK; ^6^ Department of Biostatistics University of Liverpool UK; ^7^ Department of Human Ecology Centre for Research and Advanced Studies of the National Polytechnic Institute Cinvestav (Merida) Mexico; ^8^ School of Sport, Exercise and Health Sciences Loughborough University UK; ^9^ UCSD/Salk Center for Academic Research and Training in Anthropogeny (CARTA) USA

## Abstract

**Background:**

Adrenarche involves maturation of the hypothalamic‐pituitary‐adrenal axis and increased production of dehydroepiandrosterone and its sulfate ester, dehydroepiandrosterone‐sulfate (DHEA‐S). It occurs at ages 6 to 8 in industrialized populations, marking the transition from childhood to juvenility and cognitive development at middle childhood. Studies in subsistence level populations indicate a later age (8‐9) for adrenarche, but only two such studies currently exist for comparison.

**Aims:**

To investigate adrenarcheal age among Maya girls and its association with body composition and dietary variables. We hypothesized adrenarche would occur earlier given the current dual burden of nutrition in Mexico.

**Materials and Methods:**

25 Maya girls aged 7 to 9 from Merida, Mexico using ELISAs to measure salivary DHEA‐S, standard anthropometry for height, weight, and skinfolds, bioelectrical impedance for body composition variables, as well as a food frequency questionnaire for dietary information.

**Results:**

Our hypothesis was rejected—adrenarche occurred close to 9 years. While no measures of body composition were significantly associated with adrenarcheal status, girls eating meat and dairy products more frequently had significantly higher DHEA‐S levels.

**Discussion:**

Like other populations living in ecologically challenging environments, adrenarche occurred relatively late among Maya girls. Adrenarche has been linked to measures of body composition, particularly, the adiposity or body mass index rebound, but no relevant anthropometric measures were associated, possibly because of the small sample.

**Conclusion:**

Further studies are required to illuminate how adrenarcheal variation relates to developmental plasticity, body composition, pubertal progression, and animal product consumption in other transitional populations.

## 1 INTRODUCTION

Despite adrenarche's significance in explaining early life plasticity, few anthropological studies have examined this enigmatic developmental phase (Helfrecht, Dira, & Meehan, 2017; Houghton et al., 2014). Helfrecht et al. (2017) recently estimated adrenarcheal ages for Aka foragers (9 years), Ngandu horticulturalists (9 years), and Sidama agropastoralists (8 years) in sub‐Saharan Africa by comparing dehydroepiandrosterone‐sulfate (DHEA‐S) levels in hair samples from 480 children aged 3 to 18. Adrenarcheal ages were later than those in industrialized populations (6‐8 years) (Maninger, Wolkowitz, Reus, Epel, & Mellon, 2009).

Houghton et al. (2014) compared adrenarcheal ages using salivary DHEA‐S from 419 girls of white British and Bangladeshi ethnicity growing up in Bangladesh or London (Houghton et al., 2014). Median adrenarcheal ages were around 7 years, except for first‐generation migrants to London (5.3 years). The authors suggested rapid catch‐up growth and an earlier body mass index (BMI) (adiposity) rebound might explain this earlier age.

Here, we present data from a pilot study of salivary DHEA‐S levels among Maya girls from Merida, Mexico. The Maya have experienced long‐standing marginalization and adversity, and rank among the lowest socioeconomic ethnic groups in Mexico (Azcorra et al., 2013). Despite improvements in living conditions, many children are stunted (Wilson, 2011) with recent experience of the dual burden of malnutrition (Azcorra et al., 2013; Wilson, 2011). Since adrenarcheal age is linked to timing of the BMI rebound (Remer & Manz, 1999), we hypothesized an earlier adrenarche compared to industrialized populations.

## 2 MATERIALS AND METHODS

Twenty‐five Maya girls aged 7 to 9, living with their biological mothers and attending state schools, participated (February‐August 2010) as part of a larger study on the dual burden (n = 58) (Wilson, 2011).

Anthropometrics were obtained using standardized techniques (Lohman et al., 1999). Z‐scores for height and weight were derived using Frisancho's Comprehensive Reference from NHANES III (Frisancho, 2008; Wilson, 2011). Body fat percentage and fat mass were assessed using bioelectrical impedance (Maltron BioScan 916). Body fat percentage, fat mass, and fat free mass (kg) indices were calculated using formulas validated by Lohman et al. (1999) and Ramírez et al. (2012), respectively. Birth weight was based on mothers' recall and may be biased upward (Wilson, 2011); we used the WHO cutoff of <2.5 kg for low birth weight. Mothers completed a modified version of the Pubertal Development Scale (PDS) for their daughters (Petersen, Crockett, Richards, & Boxer, 1988).

Animal product consumption, categorized into “meat” and “dairy” came from food‐frequency questionnaires administered to mothers simultaneously with anthropometric collection (Azcorra et al., 2013; Wilson, 2011). Consumption frequency was calculated, medians determined, and coded as higher/lower per week compared to the sample median. Mann‐Whitney *U* tested for associations between higher consumption and DHEA‐S levels. For meat consumption, the median, and interquartile ranges (IQR) of DHEA‐S levels for high and low frequency groups were determined.

Since DHEA‐S shows little diurnal variation, a single, morning (08:00‐12:30), stimulated saliva sample was collected in 5 mL polypropylene vials at least 1 hour after eating or drinking using flavorless gum base (Cafosa^©^, Barcelona, Spain). Samples were kept on ice until transported to the laboratory at CINVESTAV‐Merida, and then stored at −80°C until assayed using a DHEA‐S ELISA (Salimetrics®). Samples were processed in a single batch according to manufacturer instructions. ELISA absorbance was read using a Biorad microtiter plate reader at 450 nm without correction. Reproducibility was assessed by two pooled, blinded quality control samples. Intra‐assay coefficient was 19.7%, detection limits 43 and 16 000 pg/mL, respectively, and lower limit of functional assay range, 188.9 pg/mL.

Girls with DHEA‐S levels <400 pg/mL (clinical cutoff) (Havelock, Auchus, & Rainey, 2004; Houghton et al., 2014) were classified as pre‐adrenarcheal. Associations between anthropometrics and body composition were analyzed using Mann‐Whitney *U*. Age‐ and sex‐specific z‐scores for body composition were coded as binary variables: girls above the sample median of z‐scores for weight, height, and BMI‐for‐age were scored as 1, while girls equivalent or below the sample median were coded as 0. Fisher's exact test analyzed associations between being above the adrenarcheal cutoff of 400 pg/mL (“post‐adrenarcheal”), and above the sample median z‐scores for body composition variables. We also tested for associations between adrenarcheal status and 5th percentile of z‐scores for height‐for‐age (short stature/stunting), 5th percentile weight‐for‐age (under‐weight), as well as 5th, 85th, 95th percentiles of BMI‐for‐age (wasting, overweight, and obesity, respectively) (CDC, 2013).

A Fisher's exact test was used to assess whether being above the sample median for absolute fat percentage, fat mass index, and fat free mass index was associated with being post‐adrenarcheal. Overfat was defined as >85th percentile of sex‐ and age‐specific reference curves for body fat derived from British children; no equivalent exists for Maya (McCarthy, Cole, Fry, Jebb, & Prentice, 2006). Finally, we assessed birth weight and association with adrenarcheal status using the sample median as well as WHO definition of low birth weight (≤2.5 kg). Statistical analyses used Excel version 16.29.1, and SPSS version 24. Significance was *P* < .05. Means are shown with standard deviations (SD).

Girls were recruited through six primary schools and parents gave written or verbal informed consent, and children verbal assent. Ethical approval came from Durham University's Department of Anthropology Ethics Committee, Loughborough University (R09‐P145), and the Bioethics Committee of CINVESTAV.

## 3 RESULTS

Supporting Information Table 1S shows descriptive statistics for the sample. Six girls were post adrenarcheal (mean age = 9.0 ± 0.86), while 19 were pre‐adrenarcheal (mean age = 8.3 ± 0.56). Fifteen (60%) of 25 saliva samples were <188.9 pg/mL, the functional sensitivity of the DHEA‐S assay. Results from the PDS confirmed few girls were post‐adrenarcheal (Table 2S).

**FIGURE 1:**
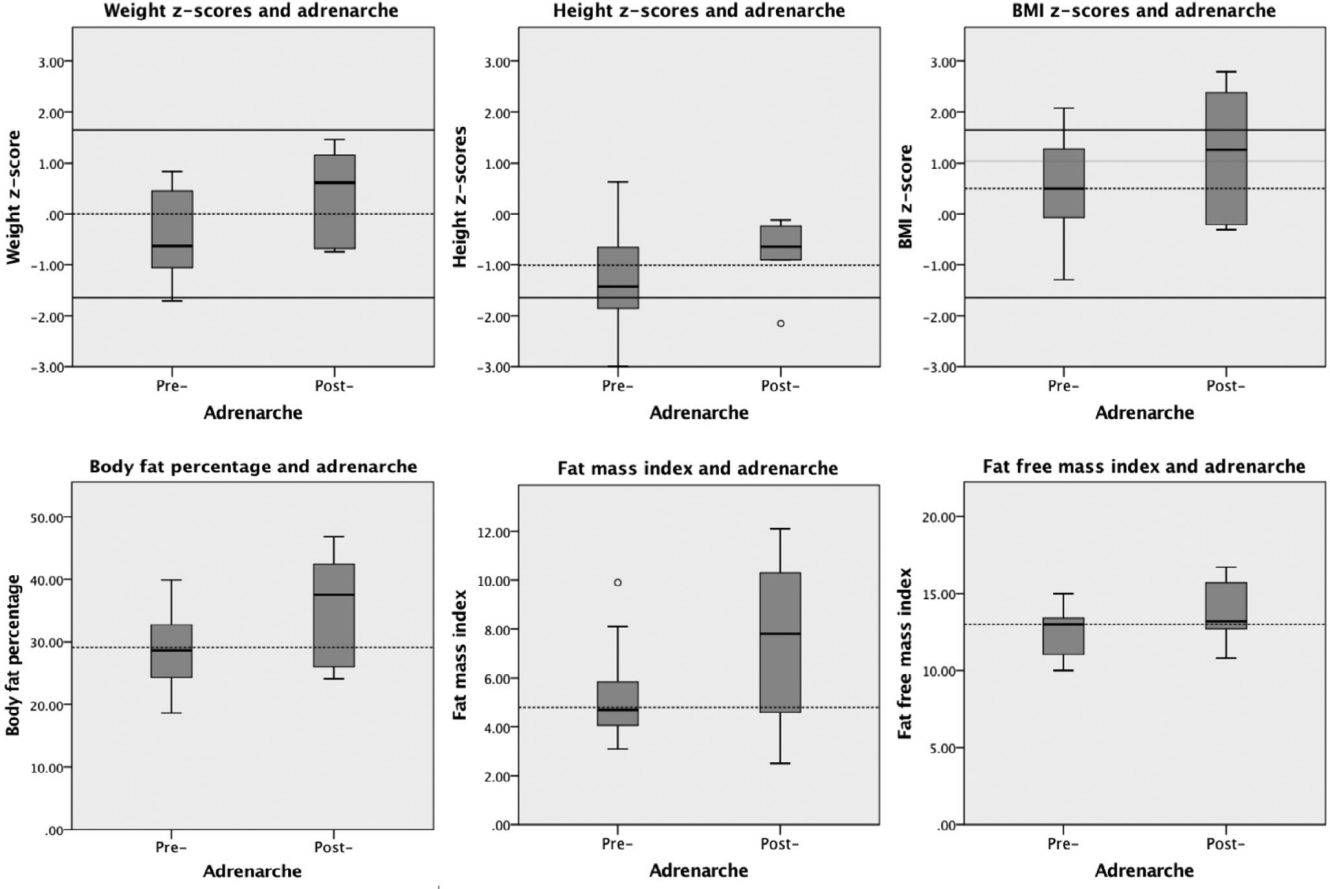
Boxplots of anthropometrics and body composition in relation to adrenarcheal status of 25 Maya girls from Merida, Mexico. The top panel displays weight‐for‐age, height‐for‐age, and BMI‐for‐age z‐scores, whereas the lower panel shows body fat percentage, fat mass index, and fat free mass index, for pre‐adrenarcheal and post‐adrenarcheal girls. The black lines indicate the CDC cut‐off points for the 5th and 95th percentile z‐scores. The dashed lines indicate the sample median for the z‐scores, body fat percentage, fat mass index, and fat free mass index, respectively

Weight was significantly associated with adrenarcheal status (U = 23.00, *P* = .030), but not z‐score median‐for‐weight, presumably because this adjusted for age. Of ten stunted girls in the sample, only one (aged 9.12 years) was post‐adrenarcheal. Neither height, measured as a continuous variable, nor being stunted was significantly associated with adrenarcheal status. More overweight (>85th percentile of BMI z‐scores) and obese girls (>95th percentile of weight‐for‐height z‐scores) were post‐adrenarcheal, but no measure relating to body weight status was significantly associated with adrenarche (Figure 1).

There were no significant associations between adrenarche and body fat percentage and fat mass, although twice as many post‐adrenarcheal girls scored higher for these indices. No clear pattern between fat free mass index and adrenarcheal status emerged. No statistic related to birth weight was significantly related to adrenarche. Using median birth weight, more pre‐adrenarcheal girls (n = 11) had lower birth weight than post‐adrenarcheal ones (n = 1).

**FIGURE 2:**
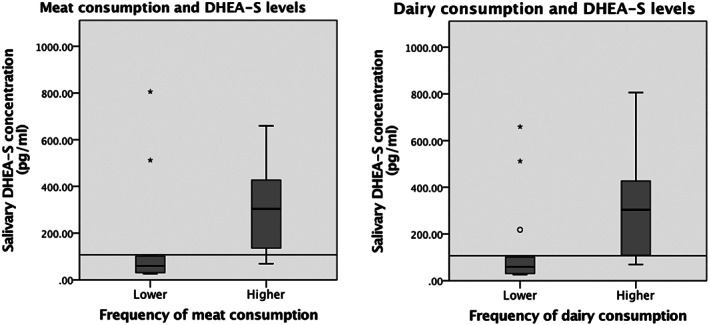
Frequency of meat and dairy consumption compared to the sample median and its effect on DHEA‐S levels in 25 Maya girls from Merida, Mexico. The black line at 107.3 pg/mL indicates the median DHEA‐S levels of the total Merida sample as measured in saliva. Animal product consumption, categorized into “meat” and “dairy,” came from food‐frequency questionnaires administered to mothers simultaneously with anthropometric collection (Azcorra et al., 2013; Wilson, 2011)

Girls with greater weekly meat consumption frequencies had significantly higher DHEA‐S (U = 29.00, *P* = .007). The median DHEA‐S level in the lower consumption group was 59.49 pg/mL (IQR: 29.99‐104.25 pg/mL) compared to 302.90 pg/mL (IQR: 123.33‐430.50 pg/mL) for girls eating meat more frequently (Figure 2). Consuming more dairy was also significantly associated with DHEA‐S levels (U = 33.00, *P* = .014). BMI was neither correlated with meat (*r* = 0.018, *P* = .934) nor dairy consumption (*r* = −0.359, *P* = .078).

## 4 DISCUSSION

Little is known about normal variation in adrenarcheal age among human populations living in diverse settings. Given increasing overweight/obesity among Maya and the temporal association of adrenarche with the BMI rebound, we hypothesized that the girls studied here would experience adrenarche earlier than those in industrialized societies. However, this hypothesis was rejected since our preliminary data suggested Maya girls reached adrenarche between 8 and 9 years, closer to ages reported for sub‐Saharan populations occupying marginal environments (Helfrecht et al., 2017). Adrenarcheal status was not significantly associated with most age‐adjusted anthropometric, standardized measurements, perhaps reflecting the small sample size. In other studies, adrenarcheal timing appears closely related to changes in body composition and energetic status (Remer & Manz, 1999), although a causal relationship between body composition and adrenarche is disputed (Bogin, in press).

Campbell (2020) suggests that, given neurological functions of DHEA‐S, rising adrenarcheal levels are crucial for cortical maturation and synaptic modification which translate phenotypically into increased competencies in middle childhood. He proposes that adrenarche might be a developmental pathway through which increased meat consumption contributed to brain expansion during *Homo* evolution. In our study here, higher meat (and dairy) consumption among Maya girls were associated with higher DHEA‐S levels.

The results here push the boundaries of adrenarcheal age later in populations exposed to more challenging ecological conditions relative to girls in well‐nourished, affluent settings. How this later transition might affect the timing of adolescence and young adulthood requires further analyses of larger cross‐sectional and longitudinal datasets. The consumption of animal products in relation to adrenarche also deserves further investigation.

## AUTHOR CONTRIBUTIONS

GB, GC, LH designed the adrenarche study protocol. FD, HW, MV, and PG designed the initial study protocol that formed the core dual burden project. FD trained HW and the fieldwork team in anthropometrics, while MV trained them in bioelectrical impedance techniques. AV and LH collected the salivary samples, and AN conducted the hormonal assays in consultation with GC. SK performed the data analyses in consultation with GB and MV. AN, GB, and SK were the main authors while all others contributed ideas and suggestions to various drafts of the manuscript. We thank the girls who participated in the study and their families, as well as the field assistants who helped in various stages of this project. Special thanks goes to Jenice Tut and Hugo Azcorra who assisted in data collection. Finally we would like to acknowledge the late Dr. Omar Zapata, who kindly stored our saliva samples before analysis.

## Supporting information


**Appendix S1:** Supporting information

## References

[ajhb23465-bib-0001] Azcorra, H. , Wilson, H. , Bogin, B. , Varela‐Silva, M. I. , Vázquez‐Vázquez, A. , & Dickinson, F. (2013). Dietetic characteristics of a sample of Mayan dual burden households in Merida, Yucatan, Mexico. Archivos Latinoamericanos de Nutrición, 63(3), 209–217. 25362820

[ajhb23465-bib-0002] Bogin, B. (in press). Patterns of human growth (3rd ed.). Cambridge, UK: Cambridge University Press.

[ajhb23465-bib-0003] Campbell, B. (2020). DHEAS and human development: An evolutionary perspective. Frontiers in Endocrinology, 11, 1–14. 10.3389/fendo.2020.00101 32194506 PMC7062714

[ajhb23465-bib-0004] CDC . (2013). Use and interpretation of the WHO and CDC growth charts for children from birth to 20 years in the United States. Centers for Disease Control and Prevention. Retrieved from http://www.cdc.gov/NCCdphp/dnpa/growthcharts/resources/growthchart.pdf

[ajhb23465-bib-0005] Frisancho, A. R. (2008). Anthropometric standards for the assessment of growth and nutritional status: An interactive nutritional reference of body size and body composition for children and adults (2nd revisied ed.). Ann Arbor, MI: University of Michigan Press. https://www.press.umich.edu/93311/anthropometric_standards.

[ajhb23465-bib-0006] Havelock, J. C. , Auchus, R. J. , & Rainey, W. E. (2004). The rise in adrenal androgen biosynthesis: Adrenarche. Seminars in Reproductive Medicine, 22(4), 337–347 Retrieved from https://www.thieme-connect.de/products/ejournals/pdf/10.1055/s-2004-861550.pdf 15635501 10.1055/s-2004-861550

[ajhb23465-bib-0007] Helfrecht, C. , Dira, S. , & Meehan, C. L. (2017). DHEAS patterning across childhood in three sub‐Saharan populations: Associations with age, sex, ethnicity, and cortisol. American Journal of Human Biology, 30, e23090. 10.1002/ajhb.23090 29226590

[ajhb23465-bib-0008] Houghton, L. C. , Cooper, G. D. , Booth, M. , Chowdhury, O. A. , Troisi, R. , Ziegler, R. G. , … Bentley, G. R. (2014). Childhood environment influences adrenarcheal timing among first‐generation Bangladeshi migrant girls to the UK. PLoS One, 9(10), e109200. 10.1371/journal.pone.0109200 25309977 PMC4195659

[ajhb23465-bib-0009] Lohman, T. , Hirnes, J. , Caballero, B. , Skipper, B. , Reid, R. , Siewart, D. , & Hunsberger, S. (1999). Body composition assessment in American Indian children. American Journal of Clinical Nutrition, 69, 764S–766S. 10.1093/ajcn/69.4.764s 10195600

[ajhb23465-bib-0010] Maninger, N. , Wolkowitz, O. M. , Reus, V. I. , Epel, E. S. , & Mellon, S. H. (2009). Neurobiological and neuropsychiatric effects of dehydroepiandrosterone (DHEA) and DHEA sulfate (DHEAS). Frontiers in Neuroendocrinology, 30(1), 65–91. 10.1016/j.yfrne.2008.11.002 19063914 PMC2725024

[ajhb23465-bib-0011] McCarthy, H. D. , Cole, T. J. , Fry, T. , Jebb, S. A. , & Prentice, A. M. (2006). Body fat reference curves for children. International Journal of Obesity, 30(4), 598–602. 10.1038/sj.ijo.0803232 16570089

[ajhb23465-bib-0012] Petersen, A. C. , Crockett, L. , Richards, M. , & Boxer, A. (1988). A self‐report measure of pubertal status: Reliability, validity, and initial norms. Journal of Youth and Adolescence, 17(2), 117–133. 10.1007/BF01537962 24277579

[ajhb23465-bib-0013] Ramírez, E. , Valencia, M. E. , Bourges, H. , Espinosa, T. , Moya‐Camarena, S. Y. , Salazar, G. , & Alemán‐Mateo, H. (2012). Body composition prediction equations based on deuterium oxide dilution method in Mexican children: A national study. European Journal of Clinical Nutrition, 66(10), 1099–1103. 10.1038/ejcn.2012.89 22805494

[ajhb23465-bib-0014] Remer, T. , & Manz, F. (1999). Role of nutritional status in the regulation of adrenarche. Journal of Pediatric Endocrinology and Metabolism, 84, 936–944. 10.1210/jcem.84.11.6093 10566631

[ajhb23465-bib-0015] Wilson, H. J. (2011). Health indicators in double burdened urban Maya children and mothers. Loughborough, UK: Loughborough University Retrieved from https://repository.lboro.ac.uk/articles/Health_indicators_in_double_burdened_urban_Maya_children_and_mothers/9605219

