# Supplementary material for: The timing of adrenarche in Maya girls, Merida, Mexico
Source: Am J Hum Biol. 2020 Jul 9;33(2):e23465. doi: 10.1002/ajhb.23465 (PMC8264844; doi:10.1002/ajhb.23465)
Supplement: Supplementary file 1 — Appendix S1: Supporting information [file AJHB-33-e23465-s001.docx]

Supplementary Tables

*Table 1S: Descriptive statistics for age, DHEA-S levels, anthropometrics, z-scores, and body composition variables of 25 Maya girls living in the city of Merida, Mexico.*

|  | **Age (years)** | **DHEA-S levels (pg/ml)** | **Birth weight (kg)** | **Height (cm)** | **Height z-score** | **Weight (kg)** | **Weight z-score** | **BMI (kg/m^2^)** | **BMI z-score** | **Body fat %** | **Fat mass (kg)** | **Fat free mass (kg)** | **Fat mass index** | **Fat free mass index** |
| --- | --- | --- | --- | --- | --- | --- | --- | --- | --- | --- | --- | --- | --- | --- |
| **1** | 9.3 | 59.49 | 2.75 | 124.7 | -1.66 | 24.85 | -1.09 | 15.98 | -0.31 | 28.66 | 8.11 | 16.74 | 10.8 | 5.2 |
| **2** | 8.12 | 75.08 | 2.2 | 114 | -1.95 | 20.8 | -0.98 | 16 | 0.05 | 23.36 | 6.06 | 14.74 | 11.3 | 4.7 |
| **3** | 8.71 | 217.9 | 3.3 | 124.8 | -1.19 | 32.45 | 0.26 | 20.83 | 1.37 | 34.58 | 9.12 | 23.33 | 15.0 | 5.9 |
| **4** | 8.67 | 107.3 | 3.2 | 124.7 | -0.71 | 27.35 | -0.14 | 17.59 | 0.54 | 30.42 | 5.91 | 21.44 | 13.8 | 3.8 |
| **5** | 8.58 | 46.5 | 2.93 | 124.4 | -0.75 | 34.2 | 0.77 | 22.1 | 1.84 | 39.89 | 11.42 | 22.78 | 14.7 | 7.4 |
| **6** | 6.95 | 48.72 | 2.25 | 103.2 | -2.89 | 17.6 | -1.03 | 16.53 | 0.5 | 26.6 | 5.16 | 12.44 | 11.7 | 4.8 |
| **7** | 8.56 | 88.72 | 2.9 | 122.7 | -1.01 | 34.8 | 0.84 | 23.11 | 2.07 | 37.18 | 14.86 | 19.94 | 13.2 | 9.9 |
| **8** | 9.03 | 69.51 | 3.75 | 128.7 | -0.61 | 33.25 | 0.36 | 20.07 | 1.17 | 29.11 | 11.78 | 21.47 | 13.0 | 7.1 |
| **9** | 9.83 | 512.3 | 3.4 | 138.9 | -0.12 | 36.5 | 0.22 | 18.92 | 0.57 | 32.9 | 11.78 | 24.72 | 12.8 | 6.1 |
| **10** | 9.68 | 452.5 | N. R.^†^ | 134.5 | -0.24 | 43.25 | 1.16 | 23.91 | 1.94 | 46.84 | 18.67 | 24.58 | 13.6 | 10.3 |
| **11** | 7.94 | 395.1 | 3.5 | 123.6 | -0.41 | 31.25 | 0.7 | 20.46 | 1.56 | 35.14 | 8.49 | 22.76 | 14.9 | 5.6 |
| **12** | 9.12 | 659.5 | 3.5 | 118.5 | -2.15 | 39.05 | 1.01 | 27.81 | 2.79 | 42.47 | 16.99 | 22.06 | 15.7 | 12.1 |
| **13** | 8.13 | 255.1 | 2.8 | 113.1 | -2.1 | 19.2 | -1.32 | 15.01 | -0.38 | 20.01 | 5.34 | 13.86 | 10.8 | 4.2 |
| **14** | 9.24 | 350.7 | 3.1 | 126.3 | -1.43 | 27.75 | -0.63 | 17.4 | 0.22 | 30.97 | 6.45 | 21.30 | 13.4 | 4.0 |
| **15** | 8.42 | 28.79 | 3.5 | 126.9 | -0.38 | 34.5 | 0.8 | 21.42 | 1.67 | 34.56 | 13.03 | 21.47 | 13.3 | 8.1 |
| **16** | 9.51 | 805.9 | 3.75 | 133.3 | -0.41 | 46.65 | 1.46 | 26.25 | 2.38 | 42.2 | 16.89 | 29.76 | 16.7 | 9.5 |
| **17** | 8 | 419.7 | 3.25 | 120.6 | -0.88 | 22.05 | -0.74 | 15.16 | -0.31 | 25.97 | 3.57 | 18.48 | 12.7 | 2.5 |
| **18** | 7.86 | 30.67 | 3.5 | 113.6 | -2.02 | 22.1 | -0.73 | 17.13 | 0.5 | 26.71 | 5.23 | 16.87 | 13.1 | 4.1 |
| **19** | 8.27 | 29.31 | 2.7 | 118.3 | -1.7 | 18.75 | -1.71 | 13.4 | -1.29 | 18.65 | 4.35 | 14.40 | 10.3 | 3.1 |
| **20** | 8.18 | 101.2 | 2.82 | 115.2 | -1.76 | 18.6 | -1.45 | 14.02 | -0.86 | 25.12 | 5.33 | 13.27 | 10.0 | 4.0 |
| **21** | 8.04 | 26.04 | 3.6 | 115.5 | -1.71 | 21.3 | -0.88 | 15.97 | 0.04 | 23.54 | 5.46 | 15.84 | 11.9 | 4.1 |
| **22** | 7.86 | 160.3 | 4.4 | 130.3 | 0.63 | 30.1 | 0.55 | 17.73 | 0.71 | 30.02 | 7.37 | 22.73 | 13.4 | 4.3 |
| **23** | 8.86 | 80.77 | 2.15 | 113.1 | -3 | 21 | -1.52 | 16.42 | -0.02 | 21.42 | 7.40 | 13.60 | 10.6 | 5.8 |
| **24** | 8.66 | 111 | 2.95 | 127.8 | -0.24 | 25.85 | -0.37 | 15.83 | -0.13 | 26.46 | 5.42 | 20.43 | 12.5 | 3.3 |
| **25** | 7.89 | 434.1 | 2.8 | 120.5 | -0.9 | 22.35 | -0.68 | 15.39 | -0.21 | 24.11 | 6.64 | 15.71 | 10.8 | 4.6 |
| **Mean**  **±SD** | **8.54**  **±0.68** | **222.65 ±220.28** | **3.13 ±0.54** | **122.29 ±8.05** | **-1.18 ±0.90** | **28.22 ±8.13** | **-0.21 ±0.95** | **18.58**  **±3.81** | **0.66 ±1.06** | **30.28 ±7.40** | **8.83 ±4.38** | **19.39 ±4.44** | **12.8 ±2.48** | **5.8 ±1.77** |
| **Median** | **8.56** | **107.30** | **3.15** | **123.60** | **-1.01** | **27.35** | **-0.37** | **17.40** | **0.50** | **29.11** | **7.37** | **20.43** | **13.0** | **4.8** |
| **SE** | **0.14** | **44.06** | **0.11** | **1.61** | **0.18** | **1.63** | **0.19** | **0.76** | **0.21** | **1.48** | **0.88** | **0.89** | **0.35** | **0.50** |

*† The mother of this participant was not able to recall the birthweight of her child.*

*Table 2S: Physical development scale characteristics related to adrenarche of 25 Maya girls in Merida, Mexico, as reported by their mother.*

| Characteristic | Stage of development  Not yet Barely Definitely started started underway  (n=) (n=) (n=) | | | Median age ± SD (years) ‘barely started’ or ‘yes’ |
| --- | --- | --- | --- | --- |
| Oily skin | 20 | 5 | 0 | 8.57±0.80 |
| Odour change | 20 | 4 | 1 | 8.70±0.50 |
| Acne | 24 | 1 | 0 | 8.71 |
| Body hair | 24 | 1 (axillary) | 0 | 9.30 |
